# MOBILIZING UNTAPPED LIVED EXPERTISE TO ASSESS ACCESSIBLE AND AGE-FRIENDLY INFRASTRUCTURES

**DOI:** 10.1093/geroni/igae098.1299

**Published:** 2024-12-31

**Authors:** Mikiko Terashima, Kate Clark, Katherine Deturbide

**Affiliations:** Dalhousie University, Halifax, Nova Scotia, Canada; Planning for Equity, Accessibility, and Community Health (PEACH) Research Unit, School of Planning, Faculty of Architecture and Planning, Dalhousie University, Halifax, Nova Scotia, Canada; Planning for Equity, Accessibility, and Community Health (PEACH) Research Unit, School of Planning, Faculty of Architecture and Planning, Dalhousie University, Halifax, Nova Scotia, Canada

## Abstract

Many rural municipalities in Canada have been experiencing steady population declines overall, while vulnerable population groups such as older adults and persons with disabilities tend to remain in their communities. With shrinking tax bases, municipalities must make smarter choices in provisions of accessible and age-friendly services and amenities. However, exact conditions and needs of accessible and age-friendly infrastructures in rural communities are increasingly becoming difficult to evaluate, as their planning departments do not have sufficient human and financial capacities to monitor them. The Planning for Equity, Accessibility, and Community Health (PEACH) Research Unit is situated in a planning school at a university in Nova Scotia, Canada. In partnership with two rural municipalities, PEACH Research Unit conducted in-person and online consultations with persons with lived expertise to develop key indicators that can inform municipalities on priority accessible and age-friendly infrastructure needs. This presentation will highlight lessons learned from our engagement processes, including considerations for accessibility and inclusive recruitment, engagement venues, and communication methods that maximize participation of a hard-to-reach group in rural communities. An example tool especially useful for our purpose was an online mapping platform, which helped the participants articulate their insights and better share stories of their experiences. It facilitated discussion beyond simply inventorying what types of infrastructure matter to them—while also soliciting their suggestions on solutions to the design of specific sites in real settings, which were often relational to surrounding environments.

